# Hospital-Acquired Bloodstream Infections in Relation to Intensive Care Unit Stays During Hospitalization—A Population-Based Cohort Study

**DOI:** 10.3390/jcm13247783

**Published:** 2024-12-20

**Authors:** Kim Oren Gradel, John Eugenio Coia, Ming Chen, Stig Lønberg Nielsen, Thøger Gorm Jensen, Jens Kjølseth Møller, Ram Benny Dessau, Pedro Póvoa

**Affiliations:** 1 Center for Clinical Epidemiology, Odense University Hospital, Kløvervænget 30, Entrance 216, Ground Floor, DK-5000 Odense C, Denmark; pedrorpovoa@gmail.com; 2 Department of Clinical Research, University of Southern Denmark, Campusvej 55, DK-5230 Odense M, Denmark; jcoia@health.sdu.dk (J.E.C.); stig.nielsen@rsyd.dk (S.L.N.); t.g.jensen@rsyd.dk (T.G.J.); 3 Department of Clinical Microbiology, Hospital of Southern Jutland, Kresten Philipsen Vej 15, DK-6200 Aabenraa, Denmark; d356042@dadlnet.dk; 4 Department of Regional Health Research, University of Southern Denmark, Campusvej 55, DK-5230 Odense M, Denmark; jens.kjoelseth.moeller@rsyd.dk (J.K.M.); ramd@regionsjaelland.dk (R.B.D.); 5 Department of Infectious Diseases, Odense University Hospital, J. B. Winsløws Vej 4, DK-5000 Odense, Denmark; 6 Department of Clinical Microbiology, Odense University Hospital, J. B. Winsløws Vej 21, DK-5000 Odense, Denmark; 7 Department of Clinical Microbiology, Vejle Hospital, University Hospital of Southern Denmark, Beriderbakken 4, DK-7100 Vejle, Denmark; 8 Department of Clinical Microbiology, Zealand University Hospital, Ingemannsvej 46, DK-4200 Slagelse, Denmark; 9 Intensive Care Unit 4, Department of Intensive Care, Hospital de São Francisco Xavier, ULSCHLO, Estrada do Forte do Alto do Duque, 1449-005 Lisbon, Portugal; 10 NOVA Medical School, NOVA University of Lisbon, Campo dos Mártires da Pátria, 130, 1169-056 Lisbon, Portugal

**Keywords:** intensive care unit, hospital-acquired bloodstream infections, prognosis, long-term outcomes, population-based, cohort study

## Abstract

**Background:** Little is known about the clinical characteristics and pathogens causing hospital-acquired bloodstream infections (HA-BSIs) in relation to an intensive care unit (ICU) stay. **Methods:** Population-based cohort study, comprising 35% of the Danish population, 2009–2016. We derived four patient groups with first-time HA-BSIs: no ICU stay during the admission (non-ICU patients) and HA-BSI acquired before, in, or after an ICU stay (before-ICU, in-ICU, and after-ICU patients). These groups were compared in relation to microbiological and clinical characteristics, including 28- and >28-day mortality. **Results:** Among 6888 HA-BSI patients, 4017, 792, 1388, and 691 were non-ICU, before-ICU, in-ICU, and after-ICU, respectively. The rates of several microorganisms differed between the patient groups, e.g., Enterococci (9.4% of non-ICU and 32.0% of in-ICU patients). The 28-day mortality was 26.3% in non-ICU, 45.0% in before-ICU, 35.6% in in-ICU, and 19.0% in after-ICU patients. The corresponding adjusted hazard ratios (95% confidence interval) were 2.10 (1.85–2.36), 1.67 (1.50–1.87), and 0.76 (0.63–0.91) (reference: non-ICU patients). There were few differences as regards >28-day mortality. **Conclusions:** We found large differences between common microorganisms and prognosis between the four patient groups. After-ICU patients had the lowest 28-day mortality despite age and comorbidity characteristics similar to the other three groups.

## 1. Introduction

Studies of hospital-acquired bloodstream infections (HA-BSIs) have frequently reported BSIs among intensive care unit (ICU) patients, but they have not looked at ICU stays during hospitalization in relation to HA-BSI episodes in general. There are numerous studies on ICU-acquired BSIs [1], but to our knowledge, no study has reported on HA-BSIs acquired before or after an ICU stay. Moreover, few studies on BSI are population-based [2], and few report long-term outcomes.

A BSI may prompt admission to an ICU. The occurrence or non-occurrence and the timing of an ICU stay during hospitalization with an HA-BSI may reveal differences in the patient’s disease journey and outcomes. An ICU-acquired BSI may be due to ICU procedures, which may also be the case for BSI detected after an ICU stay. These and other mechanisms can be assessed in population-based studies comprising ICU stays in BSI patients.

There is, thus, an unmet need to know the impact of the pathogen causing an HA-BSI according to the presence or absence of an ICU stay as well as its occurrence before, during, or after an ICU stay. This knowledge is, amongst others, important for the prescription of empiric antibiotic therapy, mortality, and length of stay.

Consequently, the present study aimed to assess the impact of the BSI diagnosis according to the place and timing of acquisition in the hospital and relation to the presence of an ICU stay, with a focus on patient characteristics, short- and long-term prognosis, and the distribution of the main microorganisms.

## 2. Materials and Methods

### 2.1. Setting

The Danish healthcare system is tax-financed and free of charge for individual patients. All BSIs are diagnosed in public hospitals covering geographically well-defined areas. The private hospitals in Denmark mainly perform elective surgery and have no acute admissions.

All Danish residents have a unique personal identification number used in all registries, which enables linkage and long-term follow-up [3].

### 2.2. Study Population

Our study comprises all adult patients (≥18 years) with HA-BSI, 2009–2016, in Region Zealand and the Region of Southern Denmark [4]. Apart from the age criterion, there were no exclusions.

We retrieved data on all positive blood cultures (BCs) from the laboratory system MADS [5] and derived BSI episodes from these data [6]. In brief, a BSI episode was defined as recognized pathogens detected in ≥1 BC set or common skin contaminants detected in ≥2 BC sets within five days. The date of the HA-BSI was defined as the date when the patient’s first positive BC was sampled.

We included each patient’s first-time BSI episode, which we linked to the Danish National Patient Registry (DNPR) [7]. The DNPR comprises codes for diagnoses, procedures, and surgery as of 1977.

We computed admissions from dates of admission from home and discharge in the DNPR. We then included all the HA-BSI episodes based on positive BCs sampled two days or later after admission [6] and retrieved dates of admission to and discharge from an ICU. For each patient, we also computed the Charlson comorbidity index (CCI) from 1977 and up to the first-time HA-BSI [8], with the exclusion of comorbidity diagnosed in the HA-BSI admission.

ICU-acquired HA-BSIs occurred ≥2 days after an ICU admission date and ≤2 days after an ICU discharge date (in-ICU patients). For the remaining admissions with one or more ICU stays, we assessed whether the HA-BSI was acquired before (before-ICU patients) or after (after-ICU patients) the admission’s first ICU stay. Consequently, the remaining patients had no ICU stays during their HA-BSI admission (non-ICU patients).

From the DNPR, we further retrieved the following organ support Nordic Medico-Statistical Committee (NOMESCO) codes during the admission’s first ICU stay (before-ICU and after-ICU patients) or in the HA-BSI ICU stay (in-ICU patients): BGDA0* (mechanical ventilation); BGDA1* (non-invasive ventilation); BJFD0* (renal replacement therapy); BFHC92*, BFHC93*, or BFHC95* (vasopressor treatment).

We further linked our study database to the Danish Civil Registration System to retrieve data on the vital status as well as dates of death and emigration, if relevant [9].

The number of ICU-free days was 0 if patients died within 28 days after the ICU admission, and it was 28 minus days in the ICU for patients surviving beyond 28 days [10].

### 2.3. BC Methods

All five departments of clinical microbiology used The BacT/Alert TM BC system (bioMérieux, Marcy l’Etoile, France), except Odense University Hospital, which used BACTEC (Becton Dickinson, Franklin Lakes, NJ, USA) until January 2011. Besides routine isolation procedures, all departments had implemented MALDI-TOF mass spectrometry by 2012.

### 2.4. Assessments of Outcomes

In the present study, the main outcomes, 28- and >28-day mortality, were computed as from the date of sampling the earliest positive BC set that defined the patient’s first BSI episode (i.e., the date of the HA-BSI).

### 2.5. Statistical Analyses

We computed contingency tables of the baseline characteristics, number of ICU stays in the HA-BSI admission, and, for in-ICU patients, in which ICU stay the HA-BSI was acquired.

We computed the number of HA-BSIs per 1000 hospital days among all patients hospitalized in the two regions [11]. For July 2013–2016, we computed numbers of HA-BSIs per 1000 ICU days, as the Danish Intensive Care Database had no data on ICU days before that [12].

For the 0–365 day period, we depicted Kaplan–Meier curves. After assessing graphically that the proportional hazard assumptions were fulfilled, we analyzed the 28- and >28-day mortality in Cox’s regression analyses with hazard ratios (HRs) and 95% confidence intervals (CIs). Follow-up ended with death, emigration, the maximum number of days (for 28-day mortality), or 31 December 2019 (for >28-day mortality), whichever came first. We applied a crude model and a model adjusted for days between hospital admission and the HA-BSI (2–4, 5–9, 10–14, 15–19, 20–24, 25–29, 30–34, 35–39, 40–44, 45–49, 50–54, 55–59, ≥60 days), age, CCI [8], sex, and the main group of microorganisms (Gram-negative, Gram-positive, fungi, poly-microbial).

The program Stata^®^ vs. 17 (StataCorp., College Station, TX, USA) was used for all analyses.

## 3. Results

### 3.1. Study Population

**Figure 1:**
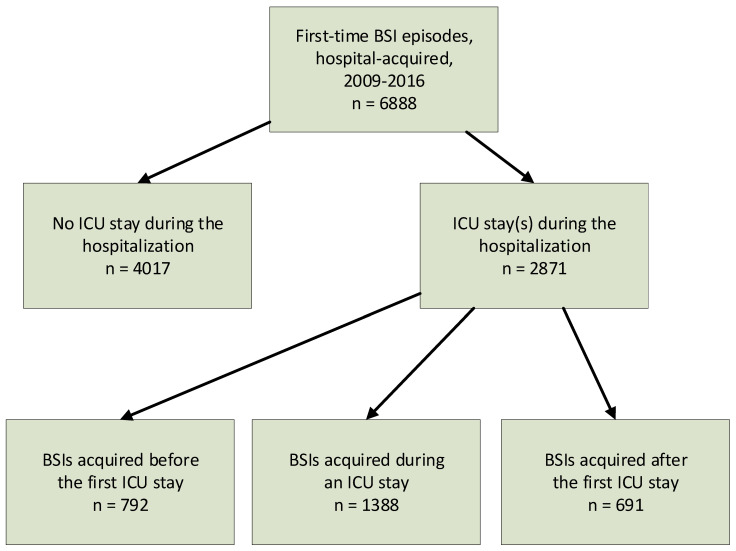
Study population. BSI = bloodstream infection.

From 2009 through 2016, 6888 adult patients had a first-time HA-BSI episode (Figure 1); 4017 (58.3%) were non-ICU, 792 (11.5%) were before-ICU, 1388 (20.1%) were in-ICU, and 691 (10.0%) were after-ICU patients.

### 3.2. General Patient Characteristics

Percent males increased from 60.8% in non-ICU to 67.9% in after-ICU patients (Table 1). The trend was the reverse for age, except that the in-ICU patients were the youngest. Non-ICU and before-ICU patients were more comorbid, whereas in-ICU patients were the least comorbid.

The picture for individual comorbidities was more heterogeneous, but several (acute myocardial infarction, congestive heart failure, cerebrovascular disease, kidney disease, leukemia, lymphoma) followed the general pattern with higher comorbidity in non-ICU and before-ICU patients (Appendix A, Table A1).

### 3.3. Distribution of Microorganisms

The biggest differences between the patient groups were seen for *Escherichia coli*, *Klebsiella* spp., Staphylococcus aureus, Coagulase-negative staphylococci (CNS), Enterococci, and fungi (Table 1). As regards Enterococci, 50.5% of the non-ICU HA-BSIs were E. faecium, whereas this was the case for 81.8% of the in-ICU HA-BSIs (Appendix A, Table A2).

### 3.4. Distribution Between Ward and ICU Days

The percentage of hospital days spent in the ICU was 50.3 for in-ICU patients, 27.8 for before-ICU patients, and 20.9% for after-ICU patients (Table 1). Days in the ICU for a patient were computed from all ICU stays during the HA-BSI admission (Appendix A, Table A3 and Table A4).

### 3.5. Incidence of HA-BSIs

The two regions had 6888 HA-BSIs and 11,530,300 hospital days among adult inpatients, i.e., 0.60 HA-BSIs per 1000 hospital days.

From 1 July 2013 through 2016, the two regions had 1639 non-ICU, 361 before-ICU, 681 in-ICU, and 308 after-ICU patients and 136,436 ICU days. This was equivalent to 12.0, 2.6, 5.0, and 2.3 HA-BSI episodes per 1000 ICU days, respectively (21.9 HA-BSI episodes per 1000 ICU days for all 2989 patients).

### 3.6. Organ Support for Patients with an ICU Stay

For most parameters, in-ICU patients had the highest levels of organ support, followed by before-ICU and after-ICU patients (Appendix A, Table A5). This trend was generally also seen for the main groups of microorganisms (Appendix A, Table A6).

### 3.7. Mortality, Length of Stay, and Time from Admission to the HA-BSI Episode

In-hospital and 28-day mortality was highest in before-ICU patients, followed by in-ICU, non-ICU, and after-ICU patients, ranging from 19% to 45% (Table 2). Among patients surviving beyond 28 days, the in-ICU patients had the lowest mortality, followed by after-ICU, before-ICU, and non-ICU patients, albeit with a lower range span (56.2–67.1%). Within each patient group, the lowest 28-day mortality was generally seen for Gram-negative HA-BSIs, followed by Gram-positive HA-BSIs, poly-microbial HA-BSIs, and fungemia.

Length of stay (median ranging from 20 to 41 days for all and from 21 to 52 days for patients discharged alive) and time from admission to the HA-BSI (median 7–20 days) differed between the patient groups (Table 2).

### 3.8. Kaplan–Meier Mortality Curves and Cox’s Regression Analyses

**Figure 2:**
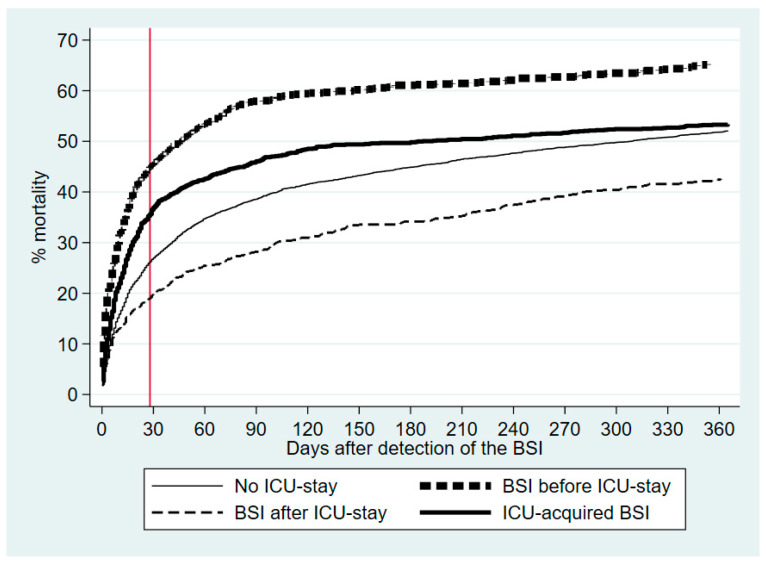
Kaplan–Meier mortality curves, 0–365 days after the bloodstream infection. The vertical red line depicts 28 days after the bloodstream infection.

Figure 2 shows the Kaplan–Meier curves for the 0–365 day mortality. After excluding seven patients who emigrated before their HA-BSI admission, the 6881 patients had 17,092 follow-up years.

The crude and adjusted Cox’s regression analyses were materially the same (Table 2). Using non-ICU patients as the reference, before-ICU patients had the highest adjusted 28-day mortality, followed by in-ICU patients. Beyond 28 days, the adjusted HRs were significantly lower for in-ICU and after-ICU patients.

## 4. Discussion

We found large differences between some common microorganisms and prognosis in relation to the four patient groups. The after-ICU patients had significantly lower 28-day mortality and fewer organ supports. Furthermore, they also had rates of several microorganisms that were intermediate between non-ICU and in-ICU patients.

For ICU-acquired BSIs, the 30-day mortality is around 40% [13], in accordance with our in-ICU patients. We speculate why before-ICU patients had higher short-term mortality than in-ICU patients did. One reason may be that BSI in the ICU may be detected earlier due to closer surveillance. Moreover, we suspect that more in-ICU patients had catheter-associated HA-BSI with CNS and *E*. *faecium* (Appendix A, Table A2), which could be treated more easily by the removal of catheters. This also applied to the after-ICU patients with their high incidence of *S*. *aureus*, CNS, and Enterococci, of which many are probably also catheter-related. From our experience, most late HA-BSIs are caused by foreign body infections or invasive procedures.

Some of the most important results of this study were the big differences between the distribution of some major microorganisms between the four patient groups, e.g., as seen for *E. coli*, *Klebsiella* spp., *S. aureus*, CNS, Enterococci, and fungi (Table 1; Appendix A, Table A2). This has direct implications for the choice of empiric antibiotic therapy as regards the occurrence of an HA-BSI in relation to an ICU stay.

In-ICU patients received more organ support than before-ICU and after-ICU patients did. After-ICU patients also had the lowest 28-day mortality, which may be biased as after-ICU patients are survivors of their first-time ICU stay, but their lower number of organ supports also indicates that they were less severely ill when admitted to the ICU.

Numerous studies have reported on the incidence of BSI in ICU patients, with 0.9% and 1.7% in the two studies having the highest numbers of patients [14,15]. Some studies have reported numbers of BSI episodes per 1000 ICU days, of which several reported around five [13,16,17,18], in accordance with our results. To the best of our knowledge, our study is the first that has assessed the reverse, i.e., incidence of ICU stays among patients with BSIs.

The after-ICU patients’ HA-BSIs were not a direct cause of their first-time ICU admission, in contrast to what it could possibly be for the before-ICU patients. We do not know the causes of ICU admission from our registry-based data, but the low number of days between the HA-BSI episode and ICU admission (median [inter-quartile range]: 0 [0–2] days; 600/792 [75.8%] with 2 days or less) for our before-ICU patients indicate that the HA-BSI was a direct cause of ICU admission in many instances. In-ICU patients constituted an intermediate group as other infections than BSIs (e.g., pneumonia) and may have contributed to their ICU admission. Our study has elucidated that the prognosis, distribution of microorganisms, age, comorbidity, and ICU-related conditions differed considerably in relation to the order and occurrence of the HA-BSIs and ICU stays. Still, as the results from the crude and adjusted analyses were materially the same, the mortality was mainly related to the patient group.

Few population-based BSI studies have reported long-term prognoses [19]. Beyond day 28, in-ICU patients tended to have lower mortality than non-ICU patients, indicating a healthy survival cohort effect among the former.

The EUROBACT2 study included 2028 patients with ICU-acquired HA-BSIs in 52 countries with 333 ICUs [1]. Although it was not population-based, it is probably the closest that can be compared to our study. A total of 63.5% were males, the median time (inter-quartile range) from hospital admission to BSI detection was 13 (8–25) days, and 28-day mortality was 37.1%, which is similar to our in-ICU patients with equivalent data of 66.5%, 13 (7–22) days, and 35.6%, respectively. Concerning the distribution of microorganisms, the main differences related to Gram-negative (59.0% in EUROBACT 2, 17.2% in our study) and Gram-positive mono-microbial BSIs (31.1%, 67.4%). Carbapenem resistance in Gram-negative microorganisms in Denmark is quite rare in contrast to the high frequencies found in EUROBACT 2. Other differences relate to our 8-year period vs. EUROBACT2 data collected in a short period and collection of data from one country in contrast to many countries with more heterogeneous data.

Our study was population-based, as patients came from two geographically well-defined regions (2,023,000 residents, 35% of Danes) [20]. The national data were highly valid [7], including ICU-related [21] and vital status data [9]. The vital status data enabled the long-term follow-up. The high number of patients enabled robust statistical analyses.

Our study, however, also had limitations. Firstly, we had little clinical data on which antibiotic treatment, microbial resistance, clinical severity, and source of infection are important. However, clinical severity was partly accounted for by the reported organ support measures. Resistance is generally low in Denmark, which has not changed since 2016 [22]. Nevertheless, resistance patterns may have some impact on the results. The broad, long-term coverage often provided to patients suspected of sepsis may select Gram-positive microorganisms. In contrast, the use of antibiotics in non-ICU settings is probably more short-term and less broad spectrum, which is less selective for drug-resistant microorganisms. As an example, Enterococci, as well as *E. faecium* vs. *E. faecalis*, were differently distributed between non-ICU and in-ICU HA-BSIs. Enterococci have high rates of resistance [23]. *E. faecalis* is generally resistant to cephalosporins, whereas *E. faecium* is additionally resistant to penicillin and carbapenems. Secondly, our data are not completely up to date. We believe that updated data would have little impact on the differences between our patient groups, and the picture of low antimicrobial resistance and the distribution of microorganisms is virtually unchanged. Thirdly, our data are only valid for Denmark and cannot necessarily be extrapolated to other countries. Finally, immortal time bias may be an important issue. As expected, the median time from admission to the HA-BSI detection differed much between the four patient groups. Adjustments for these periods did not change the results materially, and we have shown that the 30-day mortality was around 30–35% regardless of the time between hospital admission and the HA-BSI [24]. Still, we have not completely eliminated the impact of immortal time bias, and a caveat for this is warranted in the interpretation of our analyses.

## 5. Conclusions

Many factors may differ between HA-BSIs in relation to their acquisition in admissions without, before, in, or after ICU stays. BSIs and ICU stays are markers of patients, who are often older and more comorbid, but the order of these events has a considerable impact on the patients’ prognoses, especially in the short term. The better long-term prognoses for in-ICU patients are important from a public health perspective. Knowledge of the incidence of specific microorganisms in the four patient groups is important for the choice of empiric antibiotics, and we report novel and original results for after-ICU patients. The latter group of patients had the least organ support and lower 28-day mortality despite age and comorbidity characteristics similar to the other three patient groups. Our results should be assessed in other countries and settings.

## Figures and Tables

**Table 1:** Baseline characteristics for first-time hospital-acquired bloodstream infection episodes (BSIs) in adults (≥18 years).

Characteristic	No ICU Stay in Hospital Admission (n = 4017)	BSIs Acquired Before ICU Stay (n = 792)	ICU-Acquired BSIs (n = 1388)	BSIs Acquired After ICU Stay (n = 691)
	Number (%) ^a^			
Sex				
Males	2443 (60.8)	503 (63.5)	923 (66.5)	469 (67.9)
Age, years				
Median (interquartile range)	72.3 (63.2–81.2)	70.9 (62.8–7.9)	68.0 (58.6–5.1)	69.3 (60.2–76.1)
18–49	329 (8.2)	62 (7.8)	172 (12.4)	69 (10.0)
50–64	824 (20.5)	186 (23.5)	389 (28.0)	177 (25.6)
65–79	1713 (42.6)	401 (50.6)	683 (49.2)	350 (50.7)
≥80	1151 (28.7)	143 (18.1)	144 (10.4)	95 (13.8)
Charlson comorbidity index				
0 points	1075 (26.8)	225 (28.4)	559 (40.3)	230 (33.2)
1–2 points	1388 (34.5)	276 (34.8)	476 (34.3)	262 (37.9)
≥3 points	1554 (38.7)	291 (36.7)	353 (25.4)	199 (28.8)
Main microorganisms				
*Escherichia coli*	890 (22.2)	151 (19.1)	50 (3.6)	65 (9.4)
*Enterobacter* spp.	116 (2.9)	22 (2.8)	37 (2.7)	20 (2.9)
*Klebsiella* spp.	297 (7.4)	43 (5.4)	36 (2.6)	30 (4.3)
Other *Enterobacterales*	183 (4.6)	25 (3.2)	36 (2.6)	31 (4.5)
*Pseudomonas aeruginosa*	128 (3.2)	23 (2.9)	30 (2.2)	25 (3.6)
Anaerobic Gram-negative rods	100 (2.5)	15 (1.9)	5 (0.4)	14 (2.0)
Other Gram-negative bacteria	64 (1.6)	7 (0.9)	21 (1.5)	6 (0.9)
*Staphylococcus aureus*	710 (17.7)	102 (12.9)	76 (5.5)	103 (14.9)
Coagulase-negative staphylococci	319 (7.9)	47 (5.9)	283 (20.4)	106 (15.3)
*Streptococcus pneumoniae*	74 (1.8)	8 (1.0)	6 (0.4)	1 (0.1)
Hemolytic streptococci	62 (1.5)	7 (1.0)	4 (0.3)	3 (0.4)
Enterococci	376 (9.4)	115 (14.5)	444 (32.0)	111 (16.1)
Other Gram-positive cocci ^b^	104 (2.6)	17 (2.2)	10 (0.7)	5 (0.7)
Gram-positive rods	92 (2.3)	21 (2.7)	17 (1.2)	13 (1.9)
Fungi ^c^	161 (4.0)	94 (11.9)	192 (13.8)	95 (13.8)
Poly-microbial	337 (8.4)	94 (11.9)	140 (10.1)	63 (9.1)
Unidentified microorganism	4 (0.1)	1 (0.1)	1 (0.1)	0 (0)
Days in the hospital				
Days from admission to discharge	113,259	39,394	132,735	68,954
Days outside of the ICU (%)	113,259 (100)	28,437 (72.2)	66,019 (49.7)	54,543 (79.1)
Days in the ICU (%)	0 (0)	10,957 (27.8)	66,716 (50.3)	14,411 (20.9)

^a^ Except where stated otherwise; ^b^ viridans streptococci: 107/136 (78.7%); ^c^ *Candida albicans*: 290/542 (53.5%); *Candida glabrata*: 159 (29.3%); other *Candida* spp.: 91 (16.8%); not *Candida* spp.: 2 (0.4%).

**Table 2:** Mortality, length of stay, time from admission to bloodstream infection (BSI) episode, and hazard ratios with 95% confidence intervals for mortality.

Characteristic	Acquisition of the BSI Episode			
	In Admission Without Any ICU Stay	Before the ICU Stay	In the ICU	After the ICU Stay
Mortality ^a^				
0–28 days ^b^	1055 (26.3) [26.2/24.5/37.3/30.6] ^c^	356 (45.0) [42.3/44.9/52.1/46.8]	494 (35.6) [29.8/35.0/43.2/37.1]	131 (19.0) [12.6/22.3/20.0/19.4]
>28 days	1982 (67.1) ^d^ [67.7/65.7/74.3/68.7]	288 (66.2) [60.6/67.8/77.8/68.0]	501 (56.2) [52.3/55.8/64.8/54.6]	341 (61.3) [56.4/64.2/61.8/62.0]
In-hospital mortality	924 (23.0) ^e^	415 (52.4)	601 (43.3)	157 (22.7)
LOS ^f^				
Median (IQR ^g^), days	20 (12–33)	29 (15–52)	40 (22–69)	41 (25–69)
LOS, discharged alive				
Median (IQR), days	21 (13–35)	40 (26–64)	52 (30–87)	43.5 (27–72)
Time, admission-BSI ^h^				
Median (IQR), days	7 (3–14)	7 (3–13)	13 (7–22)	20 (11–34)
HRs ^i^ (95% CI ^j^) for mortality				
0–28 d mortality, crude	1 (Ref.)	1.99 (1.77–2.25)	1.44 (1.29–1.60)	0.70 (0.59–0.84)
0–28 d mortality, adj ^k^	1 (Ref.)	2.09 (1.85–2.36)	1.67 (1.50–1.87)	0.76 (0.63–0.91)
>28 d mortality, crude	1 (Ref.)	1.03 (0.91–1.17)	0.77 (0.70–0.85)	0.85 (0.76–0.95)
>28 d mortality, adj ^k^	1 (Ref.)	1.10 (0.97–1.24)	0.88 (0.80–0.98)	0.82 (0.73–0.93)

^a^ Based on 4015, 791, 688, and 1387 patients in the four groups, which differs from the numbers in Figure 1 as 7 patients emigrated before their BSI episode. Moreover, 7 emigrated after their BSI episode; ^b^ Days after sampling of the positive blood culture; ^c^ number (%) [% among Gram-negative/Gram-positive/fungal/poly-microbial BSI episodes]; ^d^ % among those alive beyond day 28; ^e^ number (%); ^f^ length of stay^; g^ inter-quartile range; ^h^ time from hospital admission to sampling of the positive blood culture; ^i^ hazard ratios; ^j^ confidence interval; ^k^ adjusted for days between hospital admission and sampling of the positive blood culture (2–4, 5–9, 10–14, 15–19, 20–24, 25–29, 30–34, 35–39, 40–44, 45–49, 50–54, 55–59, ≥60 days), age, Charlson comorbidity index (0, 1–2, ≥3), sex, the main group of microorganisms in the BSI episode (Gram-negative, Gram-positive, fungi, poly-microbial).

## Data Availability

The data that support the findings of this study are available from Statistics Denmark, but legal restrictions apply to the availability of these data, which were used under license for the current study and so are not publicly available.

## Appendix A

**Table A1:** Data for individual comorbidities in the four patient groups diagnosed before the admission with the hospital-acquired bloodstream infection.

Comorbid Disease	No ICU Stay in Hospital Admission (n = 4017)	BSIs Acquired Before ICU Stay (n = 792)	ICU-Acquired BSIs (n = 1388)	BSIs Acquired After ICU Stay (n = 691)
Acute myocardial infarction	451 (11.2)	102 (12.9)	136 (9.8)	65 (9.4)
Congestive heart failure	521 (13.0)	99 (12.5)	132 (9.5)	74 (10.7)
Peripheral vascular disease	397 (9.9)	99 (12.5)	130 (9.4)	83 (12.0)
Cerebrovascular disease	673 (16.8)	136 (17.2)	193 (13.9)	95 (13.7)
Dementia	90 (2.2)	12 (1.5)	7 (0.5)	3 (0.4)
Chronic pulmonary disease	586 (14.6)	134 (16.9)	222 (16.0)	89 (12.9)
Rheumatic disease	217 (5.4)	48 (6.1)	66 (4.8)	40 (5.8)
(Peptic) Ulcer disease	373 (9.3)	96 (12.1)	119 (8.6)	54 (7.8)
Mild liver disease	227 (5.7)	53 (6.7)	64 (4.6)	29 (4.2)
Diabetes without end-organ damage	640 (15.9)	125 (15.8)	199 (14.3)	102 (14.8)
Hemiplegia, tetraplegia	22 (0.5)	9 (1.1)	20 (1.4)	4 (0.6)
Kidney disease	403 (10.0)	87 (11.0)	85 (6.1)	45 (6.5)
Diabetes with end organ damage	375 (9.3)	67 (8.5)	101 (7.3)	67 (9.7)
Solitary tumor	1189 (29.6)	178 (22.5)	193 (13.9)	168 (24.3)
Leukemia	148 (3.7)	21 (2.7)	20 (1.4)	9 (1.3)
Lymphoma	198 (4.9)	40 (5.1)	29 (2.1)	20 (2.9)
Moderate/severe liver disease	120 (3.5)	29 (3.7)	24 (1.7)	19 (2.7)
Metastatic cancer	269 (6.7)	19 (2.4)	23 (1.7)	17 (2.5)
HIV/AIDS	3 (0.1)	2 (0.3)	4 (0.3)	2 (0.3)

**Table A2:** Distribution of *Enterococcus* spp.

Acquisition of the BSI Episode	*Enterococcus* Species			Total
	*E. faecalis*	*E. faecium*	Other Species	
Without ICU stay during hospitalization	176 (46.8)	190 (50.5)	10 (2.7)	376
Before the ICU stay	29 (25.2)	82 (71.3)	4 (3.5)	115
In the ICU	69 (15.5)	363 (81.8)	12 (2.7)	444
After the ICU stay	33 (29.7)	75 (67.6)	3 (2.7)	111

**Table A3:** Distribution of ICU stays for patients with ICU-acquired bloodstream infection (BSI) during admission.

BSI Occurred During ICU Stay	Number of ICU Stays in Admission with the BSI						Total (%)
	1	2	3	4	5	≥6	
1st	996	169	26	8	1	1	1201 (86.5)
2nd	-	127	30	4	0	0	161 (11.6)
3rd	-	-	14	1	2	1	18 (1.3)
4th	-	-	-	5	1	1	7 (0.5)
5th	-	-	-	-	0	1	1 (0.1)
Total (%)	996 (71.8)	296 (21.3)	70 (5.0)	18 (1.3)	4 (0.3)	4 (0.3)	1388 (100)

**Table A4:** Numbers of ICU stays for patients with first-time bloodstream infection (BSI) acquired in hospital before or after the admission’s first ICU stay.

BSI Acquisition in Relation to the First ICU Stay	Number of ICU Stays in Admission with the BSI					Total (%)
	1	2	3	4	5	
Before	694 (87.6)	79 (10.0)	12 (1.5)	5 (0.6)	2 (0.3)	792
After	418 (60.5)	205 (29.7)	46 (6.7)	15 (2.2)	7 (1.0)	691

**Table A5:** Organ support statistics for first-time bloodstream infections (BSIs) acquired in the hospital before, after, or in an intensive care unit (ICU) stay.

Organ Support Statistic	BSIs Acquired Before the ICU Stay (n = 792)	ICU-Acquired BSIs (n = 1388)	BSIs Acquired After the ICU Stay (n = 691)
ICU-free days			
Median (inter-quartile range)	0 (0–23)	0 (0–9)	25 (18–26)
Organ support			
Mechanical ventilation	429 (54.2) ^a^	1197 (86.2)	304 (44.0)
Non-invasive ventilation	210 (26.5)	422 (30.4)	84 (12.2)
Renal replacement therapy	150 (18.9)	461 (33.4)	74 (10.7)
Vasopressor treatment	536 (37.7)	1134 (81.8)	347 (50.2)
Number of organ supports			
0	136 (17.2)	80 (5.8)	226 (32.7)
1	211 (26.6)	157 (11.3)	203 (29.4)
2	254 (32.1)	545 (39.3)	192 (27.8)
3	158 (20.0)	457 (32.9)	58 (8.4)
4	33 (4.2)	149 (10.7)	12 (1.7)

^a^ Number (%).

**Table A6:** Number of bloodstream infection (BSI) episodes with ≥2 organ supports (vs. 0 or 1 organ support) in relation to main microorganism and acquisition before, after, or in an intensive care unit (ICU) stay.

Main Microorganism	BSI Episodes with ≥2 Organ Supports/All BSI Episodes (%)			
	All	BSI Before the ICU	BSI in the ICU	BSI After the ICU
*Escherichia coli*	121/266 (45.5)	70/151 (46.4)	33/50 (66.0)	18/65 (27.7)
*Enterobacter* spp.	56/79 (70.9)	13/22 (59.1)	35/37 (94.6)	8/20 (40.0)
*Klebsiella* spp.	65/109 (59.6)	24/43 (55.8)	32/36 (88.9)	9/30 (30.0)
Other *Enterobacterales*	52/92 (56.5)	13/25 (52.0)	26/36 (72.2)	13/31 (41.9)
*Pseudomonas aeruginosa*	46/78 (59.0)	17/23 (73.9)	22/30 (73.3)	7/25 (28.0)
Anaerobic Gram-negative rods	11/34 (32.4)	6/15 (40.0)	3/5 (60.0)	2/14 (14.3)
Other Gram-negative bacteria	26/34 (76.5)	5/7 (71.4)	17/21 (81.0)	4/6 (66.7)
*Staphylococcus aureus*	137/281 (48.8)	45/102 (44.1)	46/76 (60.3)	46/103 (44.7)
Coagulase-negative staphylococci	322/436 (73.9)	24/47 (51.1)	247/283 (87.3)	51/106 (48.1)
*Streptococcus pneumoniae*	7/15 (46.7)	3/8 (37.5)	4/6 (66.7)	0/1 (0)
Hemolytic streptococci	7/14 (50.0)	3/7 (42.9)	2/4 (50.0)	2/3 (66.7)
Enterococci	502/670 (74.9)	72/115 (62.6)	393/444 (88.5)	37/111 (33.3)
Other Gram-positive cocci	20/32 (62.5)	10/17 (58.8)	6/10 (60.0)	4/5 (80.0)
Gram-positive rods	31/51 (60.8)	10/21 (47.6)	14/17 (82.4)	7/13 (53.9)
Fungi	262/381 (68.8)	71/94 (75.5)	158/192 (82.3)	33/95 (34.7)
Poly-microbial	192/297 (64.7)	59/94 (62.8)	112/140 (80.0)	21/63 (33.3)
Gram-negatives	377/692 (54.5)	148/286 (51.8)	168/215 (78.1)	61/191 (31.9)
Gram-positives	1026/1499 (68.5)	167/317 (52.7)	712/840 (84.8)	147/342 (43.0)
Total	1857/2869 (64.7)	445/791 (56.3)	1150/1387 (82.9)	262/691 (37.9)
